# Review on Bioinspired Design of ECM-Mimicking Scaffolds by Computer-Aided Assembly of Cell-Free and Cell Laden Micro-Modules

**DOI:** 10.3390/jfb14020101

**Published:** 2023-02-13

**Authors:** Aurelio Salerno, Paolo Antonio Netti

**Affiliations:** 1 Department of Chemical, Materials, and Industrial Production Engineering, University of Naples Federico II, P.le Tecchio 80, 80125 Naples, Italy; 2 Center for Advanced Biomaterials for Healthcare, Istituto Italiano di Tecnologia (IIT@CRIB), Largo Barsanti e Matteucci, 53, 80125 Naples, Italy; 3 Interdisciplinary Research Center on Biomaterials (CRIB), University of Naples Federico II, P.le Tecchio 80, 80125 Naples, Italy

**Keywords:** CAD scaffold, micro-modules, modular tissue engineering, tissue spheroids

## Abstract

Tissue engineering needs bioactive drug delivery scaffolds capable of guiding cell biosynthesis and tissue morphogenesis in three dimensions. Several strategies have been developed to design and fabricate ECM-mimicking scaffolds suitable for directing in vitro cell/scaffold interaction, and controlling tissue morphogenesis in vivo. Among these strategies, emerging computer aided design and manufacturing processes, such as modular tissue unit patterning, promise to provide unprecedented control over the generation of biologically and biomechanically competent tissue analogues. This review discusses recent studies and highlights the role of scaffold microstructural properties and their drug release capability in cell fate control and tissue morphogenesis. Furthermore, the work highlights recent advances in the bottom-up fabrication of porous scaffolds and hybrid constructs through the computer-aided assembly of cell-free and/or cell-laden micro-modules. The advantages, current limitations, and future challenges of these strategies are described and discussed.

## 1. Introduction

The potential of tissue engineering (TE) scaffold-based approaches to repair and regenerate the functionalities of damaged and malfunctional tissues and/or organs has attracted significant research efforts in the past decades [1,2,3]. However, despite early promising results, there is a lack of representative and convincing results that demonstrate the benefit of scaffold-based approaches for clinical applications, such as bone repair and vascular implants [4,5]. For example, clinical trials demonstrated that bioresorbable scaffolds may induce a higher risk of vascular graft thrombosis and target lesion failure if compared to drug-eluting stents [5].

Native tissue growth and morphogenesis are naturally governed by a complex, dynamic cross-talk between cells and the extracellular matrix (ECM). Cells synthesize and remodel ECM components responding to signals that are both biophysical (e.g., substrate viscoelasticity and topography) and biochemical (e.g., arrays of adhesive peptides and growth factors gradients) [6,7,8,9,10,11]. In addition, ECM provides the spatiotemporal control over these signalling cues during the entire tissue regeneration process.

Computer-aided design (CAD) and additive manufacturing (AM) have revolutionized the TE field. In fact, these techniques enabled the design and manufacture of porous implantable scaffolds characterized by patient-specific size, shape and geometrical features, reliable microstructural properties, and controlled biomechanical response [2,3,12,13]. The regenerative potential of these scaffolds was considerably improved by integrating mechanisms of loading and controlled delivery of tissue morphogenic and tissue modulator molecules, such as growth factors (GFs) [14,15,16,17,18,19,20]. For instance, bioprinted drug delivery scaffolds were developed for applications such as the reconstruction of nucleus pulposus and annulus fibrosus of the intervertebral disc [21] and the repair of the regional architecture of osteochondral tissue [22].

Among the different CAD-AM processing techniques suitable for manufacturing hierarchical scaffolds towards biologically functional tissue analogues, robotic dispensing is the most used. This technique uses three main components: a temperature controlled dispensing system, a micropositioning system, and a supporting platform [23]. Robotic dispensing is a material transfer process that extrudes high viscosity bioinks from a syringe either by pneumatic extrusion, by piston-driven extrusion, or screw-driven extrusion, onto the supporting platform. Extruded bioinks are often filaments of biomaterials, such as hydrogel or thermoplastic polymers, loaded with cells and/or bioactive molecules, that are deposited following customized layer-by-layer architectures [23]. Robotic dispensing can also be combined with automated micro-tissue assembly technologies, such as tissue spheroids and/or cell laden microbeads, to build hybrid hierarchical bioconstructs, and to direct the 3D bioassembly of micro-tissues in complex, anatomically-shaped 3D scaffolds [24].

Several reviews discuss the recent progress of bioprinting in TE scaffold design and fabrication. Most of these works focused their attention on techniques such as extrusion printing and VAT polymerization [25,26]. In contrast, reviews describing current advances of ECM-mimicking scaffolds, and how the CAD-AM of cell-free and cell laden modular tissue units can be used to meet these challenges, are scarce. The aim of this work is to review the state of the art of CAD-AM processes based on the use of modular tissue units, such as cell free and cell laden microspheres and tissue spheroids, alone or in combination with robotic dispensing, towards the manufacture of advanced scaffolds for TE application. The review started with the description of the most important ECM-mimicking scaffolds design aspects, spanning from the architectural features, such as pore distribution and orientation, to scaffolds biomechanics and drug delivery, both passive and stimuli controlled. Then, the work on the latest advances on CAD-AM processes applied to micro modules positioning and assembly is described and discussed, highlighting the advantages, limitations, and future perspectives of their use in the TE field.

## 2. Current Advances of Synthetic ECM-Mimicking Scaffolds

**Figure 1:**
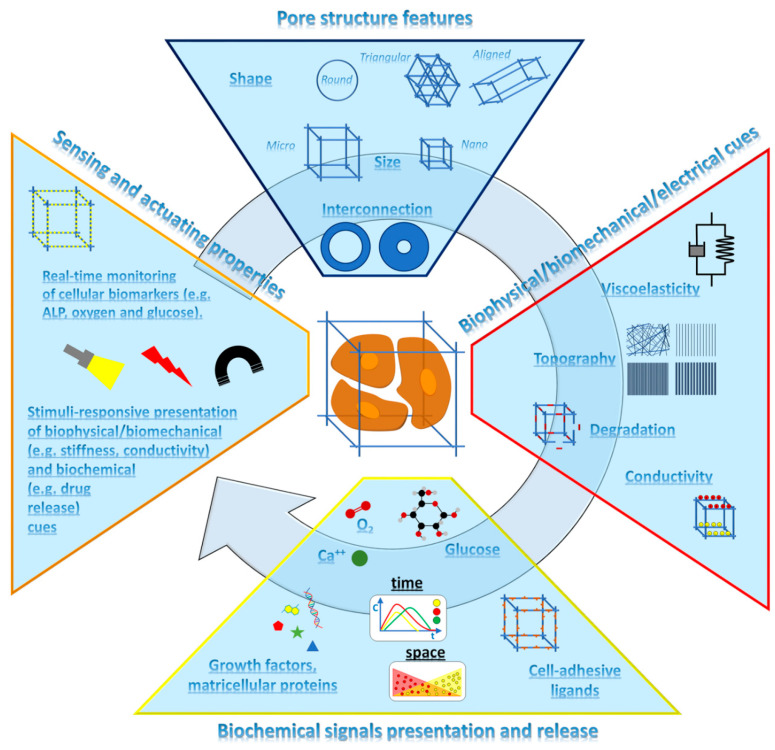
Synthetic ECM-mimicking scaffold features. Multifunctional scaffolds should provide cells with proper microenvironmental features by displaying arrays of biophysical properties and biochemicals with an on-demand logic following the evolution of new tissue morphogenesis. Clockwise, from top: scaffold architectural features, namely morphology, pore structure and size, must support cell adhesion and organization into the scaffold space to mimic the organization and structure of native tissues. Cell orientation, motility, adhesion, and differentiation can be modulated by a specific surface stiffness and micro- and nano-topography. The degradation of scaffold structure controls the space available for cell migration and tissue growth, while electrical properties are directly correlated to the orientation and growth of aligned tissues, such as nerve and muscles. Biochemical signals, such as GFs and cell adhesive ligands, must be presented with the correct conformation to elicit the desired biological response. By incorporating within scaffold material either micro- or nano-carriers loaded with bioactive peptides, the time and space evolution of these molecules can be programmed to enhance, among others, tissue vascularization and ECM deposition. The 3D structure of scaffolding materials also serves as a sensing platform for the on-time monitoring of cellular metabolic activity, and as an actuating system to promote and guide correct cell and tissue morphogenesis.

In the last decades, huge efforts have been devoted by the TE community to study and elucidate the effect of scaffolds’ properties on tissue regeneration. These properties are highlighted in Figure 1 and divided into four main groups: pore structure features, biophysical/biomechanical/electrical properties, biochemical signals presentation and release, and, finally, sensing and actuating properties.

In native tissues, cells and ECM are organized into hierarchical three-dimensional (3D) structures that define tissue composition and shape and regulate cell/ECM distribution and interaction. The porous structure of the scaffold, namely overall porosity, pore size and shape, and pores spatial distribution, must be optimized depending on the tissue to be regenerated in order to guide cell behaviour and new tissue growth [27,28,29,30,31,32]. Conventional processes, such as gas foaming and phase separation, were used to fabricate porous scaffolds mimicking the trabecular structure of spongy bone and the nano-fibrous morphology of collagen fibres of native ECM [28,29]. A higher interconnected porosity degree provides a higher effective permeability, which may better promote the transport of fluids, such as nutrients and metabolic wastes, necessary for cell survival and biosynthesis in 3D [27,28]. For bone regeneration, large pores, with sizes in the 100–400 µm range, were designed for 3D cellular colonization and proliferation, while smaller, tens of microns pores were created to enhance fluids transport [28,29]. The addition of channels in a porous scaffold can also promote cell growth and rapid vascularization, thus leading to better outcomes in new tissue formation. Besides, for the regeneration of tissues featuring oriented architectures, such as nerve, muscle, tendon, ligament, and teeth, scaffolds with aligned pores are needed to direct cell alignment and migration [13,33,34,35]. CAD-AM techniques have revolutionized TE fabrication processes as they use medical imaging combined with virtual models and automated layer-by-layer manufacturing to control the composition and structure of porous scaffolds to meet patient-specific requirements [25,26]. Composite scaffolds made of biodegradable polyesters, such as poly-lactide-co-glycolide (PLGA) and poly(e-caprolactone) (PCL), loaded with different types of inorganic osteoinductive fillers were developed to mimic the native bone and osteochondral tissues architecture [36,37].

Anchorage dependent cells sense and respond to properties of the ECM, namely substrate topography, viscoelasticity, degradation, and conductivity (Figure 1). Cell adhesion to a biomaterial scaffold is a complex process that is mediated by specific proteins, the integrins. This binding originates a force on the cytoskeletal components of the cells and, concomitantly, induces the activation of signalling pathways and messengers [38,39]. The composition and structure of adsorbed proteins, that mainly depend on substrate chemistry, energy, topography, and hydrophilicity, influence cell adhesion and spreading [39]. Nanometric and micrometric size scale topographies were also studied to control the distribution and conformation of cell-adhesive proteins, such as fibronectin, collagen, and laminin [38]. In fact, it was demonstrated in vitro that surfaces patterned with microgrooves promoted stem cells differentiation into aligned nerve cells [40], adipogenic cells [41], and osteoblasts [42]. For instance, the neuronal differentiation rate and neurite outgrowth of stem cells cultured on micropatterned polydimethyl siloxane surfaces decreased when micropattern widths approached soma diameter due to unfavourable topographical constraints [40]. The topographical characteristics of the pattern also affect mesenchymal stem cell differentiation through the modulation of focal adhesions [42]. By increasing the width of ridges in the 2–15 µm range, cell adipogenic differentiation increased [42]. On the contrary, cells seeded onto smaller ridges appeared elongated, characterized by fewer and smaller focal adhesions, and differentiated towards osteoblastic lineage cells [42]. The adsorption and conformation of cell-adhesive proteins also depend on surface hydrophilicity. It was reported that moderately hydrophilic polyethylene irradiated with plasma and grafted with polyethylene glycol (contact angle ~60°) provided optimal cell adhesion. On the contrary, highly hydrophilic surfaces reduced or, in some cases, prevented the adsorption of proteins, while proteins adsorbed onto highly hydrophobic materials are too rigid, may degrade, and hamper cell adhesion [43]. The importance of the adsorption of specific proteins, such as fibronectin and vitronectin, on cell adhesion and tissue growth was also studied in 3D porous scaffold cultures [44].

Surface topography is not the sole parameter that was found to affect cell behaviour. In native tissues, living cells are continuously subjected to mechanical stimuli arising from the surrounding cells and ECM. The intracellular molecular process through which such physical cues are transformed into a biological response, named mechanotransduction, is an intricate balance between pulling forces actuated by the actin fibres on the adhesion plaques, and the mechanical reaction of the supporting material [45]. Most importantly, cells respond to this mechanical stimulus by inducing local changes in ECM composition and mechanics that, in turn, affect the behaviour of neighbouring cells [46,47,48]. The elucidation of the molecular mechanisms by which the cell perceives and transforms the mechanics of the ECM has become the subject of intense investigation [47]. The integrin-based focal adhesion kinase (FAK) is one of the most important signalling pathways that transmits ECM stiffness to cells. Once activated, this pathway regulated transcription factors that translocate into the nucleus in response to microenvironment mechanics, and control cellular response [46]. Soft collagen functionalized gels (elastic modulus lower than 2 kPa) directed mesenchymal stem cell adipogenic differentiation, whereas stiffer gels (elastic modulus in the 30–50 kPa range) induced osteoblast differentiation [46]. Nevertheless, cells are sensitive not only to the stiffness of the supporting material, but also to the energy required to deform it [45]. As a direct consequence, higher deformation energy polydimethyl siloxane (PDMS) substrates promoted more robust fibroblasts and osteoblasts cytoskeleton assembly to counterbalance the resistance of the substrate, with the consequent cell stiffening 1 increase [45].

The rapid increase in the molecular understanding of the dynamic cell/ECM crosstalk in native tissues has inspired researchers to investigate the development of smart synthetic hydrogels, enabling cells to modulate the degradation via the release of matrix metalloproteinases [49,50,51,52,53]. By adding bioactive peptides within the poly(ethylene glycol) (PEG) hydrogel structure, researchers modulated GFs release and promoted cell migration and recruitment [50,51,52]. Among the most important cell-signalling biomolecules for tissue development and repair, there are adhesive peptides, such as arginylglycylaspartic acid (RGD) [54], intracellular signalling polypeptides, such as GFs [14], genetic material, namely DNA and RNA [55,56], as well as antibiotic, anti-inflammatory, or differentiation agents [57]. These biomolecules can be loaded into the scaffolds by direct blending with scaffold material before the bioprinting step, or by surface adsorption onto the pores of the scaffold [58,59]. Spatial and temporal gradients of these cues are often found in vivo to aid directional migration through tissue during wound healing, immune response, angiogenesis, and embryonic development [60,61]. Protein gradients exist in two forms in vivo: diffusible, where proteins diffuse away from a source, or substrate-bound, where the cues are fastened either to cell surfaces or to the surrounding ECM [62]. Chemotaxis and haptotaxis are defined as the directional cell migration in response to a gradient of soluble or substrate-bound cues, respectively. It is worth noting that some studies speculated that the mechanism of action of chemotactic gradients depends on protein adsorption to a surface to form a haptotactic gradient [62]. Multiple 2D and 3D models investigated the effect of cue gradients on the directional cell migration and outgrowth [62,63,64,65]. In recent works, biomimetic fibrous scaffolds featuring controlled gradients of chemotactic molecules were successfully engineered to repair nerve defects after in vivo implantation [64,65]. PCL scaffolds featuring bone-tissue osteon architecture were prepared by the extrusion printing of bioink loaded with 1.5 × 10^7^ mL^−1^ BMSCs and/or 10 µg mL^−1^ bone morphogenetic protein to enhance construct vascularization [66]. In another study, VEGF-loaded bioink was printed in the scaffold centre to stimulate blood vessel ingrowth, while RGD-loaded osteoinductive bioink was printed in the periphery to promote bone growth and implant integration with surrounding tissue [9]. A similar strategy was proposed by Hao and co-workers that adapted 3D printing technology to co-encapsulate chemokines and chondroinductive molecules within biomimetic PCL composite scaffolds for regenerating the meniscus [67]. Although 3D scaffold bioactivation through the incorporation of diffusible and/or bounded cues is widely used to enhance tissue regeneration, such a strategy can be affected by poor stability and the potential immunogenicity of biomolecules, as well as the difficulty to direct biomolecule delivery to cell population targets to enhance their therapeutic activity and minimize the overdosage toxic effect. Therefore, nano and microcarriers or hydrogel bioink are required as encapsulation systems to protect biomolecules against possible thermal and/or chemical inactivation, and to enhance their therapeutic potential [68,69].

The integration of molecularly imprinted materials responding to internal (e.g., cell-secreted protein degradable hydrogels) and external stimuli (e.g., temperature, electric and magnetic fields, light-responsive systems) in 3D CAD-AM scaffolds may open new avenues to control the presentation of biomolecular signals following a cell-demanding mechanism, and to recapitulate the cell/ECM dual reciprocity interaction [70]. Loading magnetically responsive particles within scaffolds was used to trigger the release of biomolecules, such as GFs, to stimulate cells proliferation and differentiation without affecting matrix degradation [71,72]. Magnetically activated scaffolds were recently fabricated to promote in vivo vascular recovery by macrophages recruiting, followed by the proliferation of mouse aortic vascular smooth muscle cells and rapid reendothelialization [73]. Light is a non-invasive stimulus that enabled high spatial resolution and the temporal control of drug release [17]. 3D printed hydrogel patches decorated with photoactive and antibacterial tetrapodal zinc oxide microparticles were developed as light-activated scaffolds for wound healing [74]. Light-actuated drug delivery was also achieved by the reversible cis-to-trans conformational change of azobenzenes once exposed to UV and visible light irradiation [75,76]. Previous cited works demonstrated that the process of scaffold-mediated tissue growth can be orchestrated by the proper design and combination of different scaffold features. However, tissue regeneration occurs over timescales ranging from seconds to months. Therefore, there is the need for the on-line monitoring of cell metabolisms, morphology, growth, and proliferation status over time, and within the different sections of the bioconstructs. This will allow for understanding which processes the cells need help with, and adjust scaffold mechanical stimulation, degradation, and release to meet cell/tissue requirements [77,78].

## 3. CAD Assembly of Cell-Free and Cell Laden Micro-Modules to Meet ECM-Mimicking Scaffolds Challenges

The design and fabrication of the next generation of multifunctional ECM-mimicking scaffolds require the capability to program the physical location and lifetime of topographical and biomolecular signals in a coordinated way during the entire process of neo-tissue formation. This challenging goal requires breaking down the complexity of 3D scaffold building by using CAD-AM bottom-up strategies. The tenet of these bottom-up strategies is to obtain complex patient-specific scaffolds through the assembly of micro- and nano-sized objects encoding active biochemical cues to recreate cell niche compositions and structures [79,80]. As previously discussed, bioprinting processes commonly use filament extrusion or bioresin photopolymerization techniques to build 3D porous ECM-mimicking scaffolds. To date, the main limitations of these approaches are the possible detrimental effects of shear stresses and light on encapsulated cells and biomolecules, together with the limited range of mechanical properties of cell-laden hydrogel materials. Most notably, important advances in materials science and processing are still required to increase the degree of precision of bioscaffold design features, down to the sub-cellular size scale.

**Figure 2:**
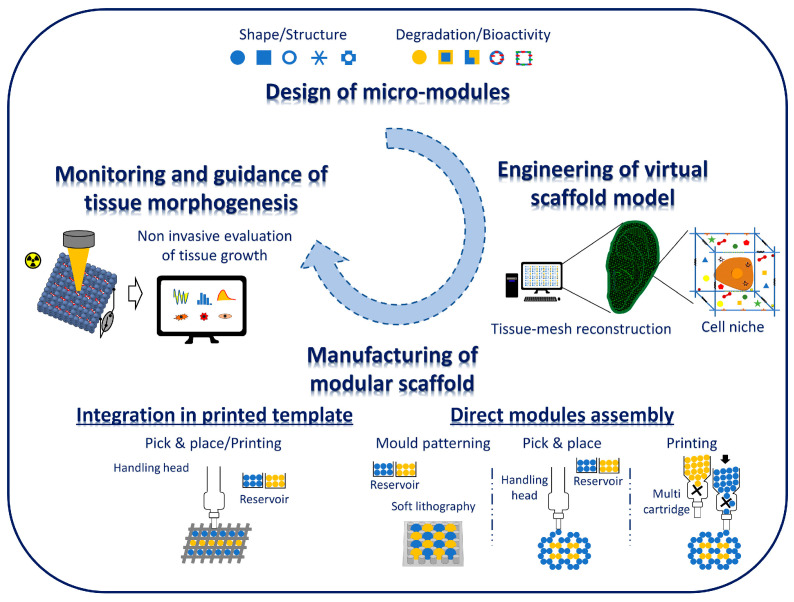
Challenges of bottom-up ECM-mimicking scaffolds design, engineering, and fabrication. Design relevant properties of micro- and nano-building block modules encoding a specific function; engineer a virtual scaffold model by using advanced numerical procedures able to match the spatial arrangement of building blocks with any predefined complex molecular and structural microenvironment. Manufacture bottom-up scaffolds by modules printing and/or micro- and nano-positioning. On-time monitor and guide cells and tissue morphogenesis. The integration of these platforms would allow simultaneous but almost independent control of multiple structural features and bioactive signals together with the evaluation and possibly the stimulation of functional tissue growth.

Modular strategies that use CAD in combination with a micro-metric size module assembly promise to address this challenging goal by improving the spatial and temporal control of cells, biomaterials, and biomolecules. The basic aspects of these approaches are highlighted in Figure 2. These include (i) the design and validation of the basic properties (i.e., material/drug/cell composition, morphology, and structure) of the building blocks; (ii) the engineering of the virtual scaffold model of the bioscaffold, from cell niche up to tissue structure; (iii) the choice of the processing techniques to manufacture the final scaffolds; and (iv) the on-time monitoring and optimization of new tissue growth.

**Figure 3:**
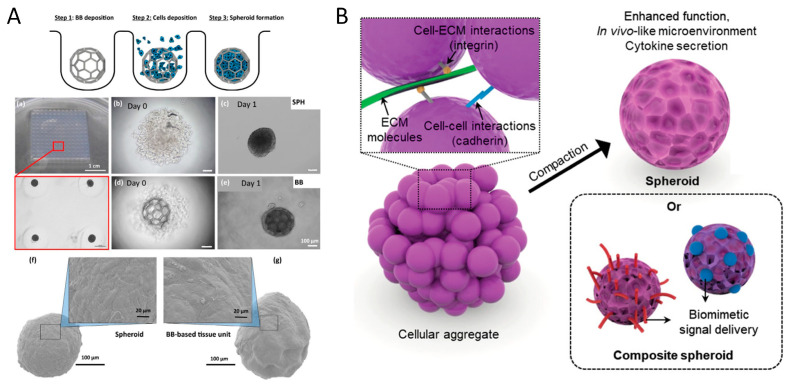
Microscaffolds and tissue spheroids micro-modules. Cell-laden micromodules can be achieved by seeding and culture of cells onto highly interconnected microscaffolds or by engineering multi-cellular spheroids. (**A**) Microscaffolds provide cells with adequate shape and resistance to mechanical stresses during handling (e.g., printing). Furthermore, microscaffolds may allow to achieve proper drug release during cell expansion and tissue development. (**B**) Tissue spheroids are formed as a 3D cellular structure with dense cell-cell/cell-ECM interactions. Spheroids can be shaped by mould and maximize cell delivery into the scaffolds. (**A**), reproduced with permission from [92]; (**B**) reproduced with permission from [93].

The fabrication of miniaturized smart nano- and micro-systems and their use to build assembling/disassembling niches for cells laboratories must be supported by the integration of the most advanced sciences of materials synthesis, drug/biomaterial conjugation and patterning. These building blocks can be manufactured in the form of microspheres, either solid or porous, hydrogels, and fibres, depending on cell source and tissue architecture requirements [81,82,83,84]. Microfluidic emulsion is one of the most investigated techniques for the high-throughput production of monodisperse nano and microparticles featuring pre-defined composition, shape, and drug loading capability [85]. Microspheres with a narrow and controllable diameter distribution and interconnected porosity can be obtained by fluidic emulsion and gelation processes [81,82,86,87,88]. Porous PCL microspheres with a bioinspired trabecular structure that supported in vitro the adhesion, growth, and biosynthesis of human dermal fibroblasts were fabricated by using polyethylene oxide as porogen [81]. Alginate-gelatin microspheres with varying stiffness, stress-relaxation, and viscoelasticity were obtained by controlling the composition of the starting co-polymer [85]. Microfluidic can be conveniently combined with the UV-lithography technique for continuously synthesizing microparticles featuring a variety of different shapes and sizes [89,90]. Recent advances in micro/nanotechnology have also allowed for the fabrication of microparticles made of thermoplastic polymers with uniform sizes and well-defined shapes and compositions, providing new building block libraries for modular TE and load bearing applications [91]. As shown in Figure 3, micro-modules can incorporate cells (microscaffolds), or may be achieved by the direct assembly of cells (tissue spheroids) [92,93]. Both modules can be used to build 3D bioconstructs for tissue regeneration, each with pros and cons. Cell laden micro-scaffolds and tissue spheroids are considered important components for in vitro cell handling and expansion [94]. Additionally, each cell type requires individualized culture conditions and scaffold components to maintain and prolong the specific cellular phenotype [94]. Therefore, it may be possible to achieve specific zonal cell/tissue heterogeneity through the proper integration of these modules within 3D bioconstructs [24]. Microscaffolds can be designed with proper porosity architectures to enhance cell adhesion and colonization [81,82,86,87,88,92]. By using hydrogel materials for microscaffold fabrication, mechanical stimulation can be optimized to control single cell biosynthesis and differentiation [95]. Multicellular spheroids are formed by the self-assembly of cells suspended in biological fluids, and therefore tissue spheroid methods maximize cell delivery, cell-cell interaction, and enhance cell viability [92].

The random assembly/compaction of cell-free and/or cell-laden modules by sedimentation, followed by sintering, was the first attempt used to obtain up to a centimetre sized scaffold and bioconstruct. Building blocks sintering was obtained by chemical reaction, physical reaction, cell-cell interaction, and external driving force [96]. Bone tissue scaffolds were obtained by heat or the chemical sintering of thermoplastic polymeric microspheres, such as PCL and PLGA [13,97]. Biological sintering, such as that modulated by cells/cells and cells/ECM interlocking, may allow for the better preservation of cellular viability and, therefore, was used to obtain hybrid cell/material constructs from cell laden modules [79,88,98,99]. Nevertheless, bio-constructs prepared by the random assembly of these modules rarely replicate the hierarchical architecture of native tissues, and therefore new advanced CAD approaches were recently proposed to fabricate bioconstructs based on in silico pre-defined patterns. As shown in Figure 2, the approaches that can be used to manufacture modular scaffolds can be classified in two main groups. The first group includes technological advances towards direct patterning of single modules into the desired geometry. These include extrusion-based bioprinting, pick and place bioprinting, and mould patterning. Extrusion-based bioprinting is the most versatile and easy approach for creating 3D modular scaffolds and bioconstructs. However, the bioprinting of cell-laden microspheres and tissue spheroids requires a slurry paste for extrusion made of modules loading inside proper bioinks [100,101]. Tan and co-workers prepared a bioink composed of cell-laden PLGA porous microspheres with a thin encapsulation of agarose-collagen composite hydrogel [102]. The porous microspheres were designed to promote cell adhesion and proliferation, while the hydrogel coating facilitated the delivery of cell-laden microspheres, and their fast gelation upon printing on top of the cold build platform. A similar strategy was used to print MSCs-laden polylactic acid microcarriers, obtained via static culture or spinner flask expansion, in gelatin methacrylamide-gellan gum bioinks [103]. Bioprinting of microgels was also widely used to fabricate large viable constructs with percolating interstitial space following in silico designs [104,105,106]. Synthetic microgels with varying degrees of degradability were assembled with or without encapsulated cells, by particle jamming and extrusion printing, and semi-orthogonal chemical cues were employed to tune the void fraction in printed scaffolds [104]. The high-throughput direct encapsulation of cells within printable microgels scaffolds with different void fractions provided unprecedented spatiotemporal control over the mechanical, topographical, and geometric cues necessary for the proper maturation of printed constructs [104]. Cell-laden microgels can be directly assembled into well-defined 3D shapes and structures under low-level ultraviolet light [105]. To achieve a higher mechanical response, an ink composed of microgels swollen in a monomer-containing solution was followed by a post curing step [106]. Cell-laden module fabrication and printing can also be achieved in a single step by using a supporting hydrogel [107]. This approach may overcome the previous steps of in vitro cell seeding and culture before bioprinting.

**Figure 4:**
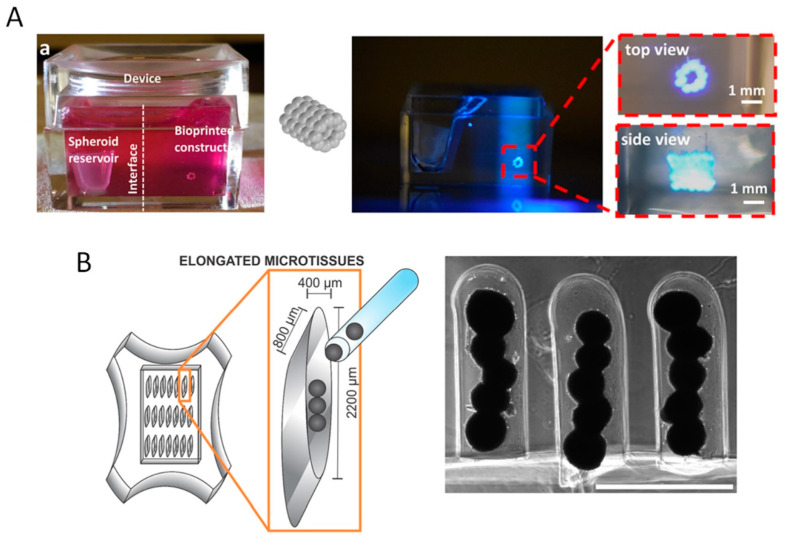
Tissue spheroids patterning by (**A**) aspiration-assisted freeform bioprinting ((**a**) The bioprinting setup, where a box was filled with the yield-stress gel in one compartment and cell media in the other) and (**B**) mould patterning. In the aspiration-assisted freeform bioprinting, the aspiration force was used to pick up spheroids from the spheroid reservoir and transfer them into the yield-stress gel one by one following five layers of circles forming a cylinder. In (**B**), 3D spheroids were generated using non-adhesive agarose gels to guide self-assembly, and subsequently transferred to non-adhesive troughs. (**A**), reproduced with permission from [109]; (**B**), reproduced with permission from [116].

Different strategies have been proposed to manipulate nano- and micrometre size scale building blocks following a virtual scaffold model without the need for bioinks. The aspiration-assisted bioprinting technique, that belongs to the pick and place techniques (Figure 2), represents a promising approach to achieve the precise control over the modules’ positioning in the 3D space [108,109]. This process allows for manipulating a wide range of modules, spanning from cell free and cell-laden microspheres to tissue spheroids, with dimensions in the range of 10^2^ to 10^3^ micrometres. Furthermore, the aspiration-assisted bioprinting of tissue spheroids in self-healing yield-stress gels was developed to manufacture freeform shapes and the self-assembly of human mesenchymal stem cell spheroids (Figure 4A). Most notably, aspiration bioprinting enabled the improvement of the positional accuracy and precision offered by other spheroid bioprinting approaches while reducing the detrimental effects on spheroid viability and tissue damage [100,108,109]. Aspiration assisted bioprinting was also proposed for the manipulation of polymeric microspheres for 3D scaffolds fabrication [108]. It is worth noting that bioconstructs prepared by extrusion bioprinting with hydrogel inks or by direct printing of tissue spheroids possess a limited capability to sustain mechanical stresses for load bearing TE applications. This is due to the inherent limitation of materials (e.g., hydrogel bioinks) and due to the aggregation mechanism, that often involves cell/ECM bridges formation. The use of mould patterning or the integration of tissue modules within the skeletal structure of a printed thermoplastic polymer may improve the capability of the scaffolds to sustain mechanical loading. For instance, mould patterning was used to obtain cell-free microsphere-sintered TE scaffolds featuring in silico defined microarchitectures [13,110,111]. The developed approach used a replica moulding technique to create arrays of pillars onto PDMS mould that can be used to fix the position of microspheres into a well definite layered configuration [13]. Microspheres sintered layers can be achieved by heat or solvent sintering and, subsequently, the layers can be stacked to build 3D scaffolds. The alignment of the microspheres and the strong bonds induced by the sintering steps enabled the achievement of stiff scaffolds, and proper tissue integration in vivo [110,111]. Although the mould patterning approach lacks significant automation, this process is highly versatile in terms of materials and design features. Indeed, by the proper combination of building blocks with different sizes, compositions, and functionalities, it may allow for using the vast library of functional building blocks available in literature to build 3D scaffolds capable of triggering, for instance, biomolecule release following a cell- and tissue-demanding strategy. Self-assembly techniques based on gravity sedimentation, mechanical vibration and capillary force were also proposed to obtain large 3D porous scaffolds with hexagonal close-packed configurations [112]. Recently, flexible polymeric masks comprised of patterned openings or textured surfaces were implemented to direct microparticles deposition onto various surfaces [113,114]. Mould patterning was also applied to cell-laden microscaffolds to build fully vascularized bio-hybrids with the micro-metric size scale control of blood vessels growth and orientation [115]. The process involved the direct seeding of human dermal fibroblasts and human vascular endothelial cells onto patterned microscaffolds until the formation of a cell/ECM sintered bioconstructs. This way, it was possible to study of the role of microscaffold configuration on cells growth and new tissue morphogenesis, finally demonstrating that bioconstruct vascularization strongly depends on building block spatial patterning [115]. In another work, the mould patterning approach was used to obtain scaffold-free 3D cardiac microtissue spheroids comprised of cardiac myocytes and/or cardiac fibroblasts (CFs) and they were used as building blocks to form larger microtissues with different cellular spatial distributions (Figure 4B) [116].

**Figure 5:**
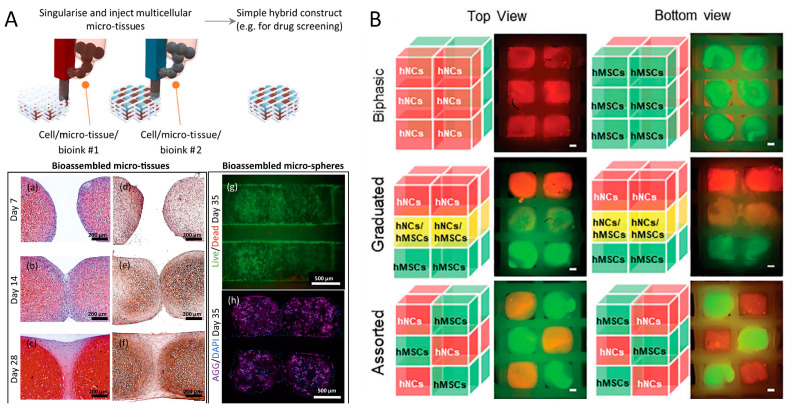
Micromodules patterning within the pores of a custom-made 3D printed thermoplastic cage. (**A**) Automated 3D bioassembly platform capable of fabricating hybrid constructs via a multistep bottom-up bioassembly strategy. The bioassembly system consisted of a fluidic-based singularisation and injection module that delivers individual micro-tissues to an injection module for insertion into precise locations within a 3D plotted scaffold (sections of assembled micro-tissues and associated tissue fusion in adjacent culture over 28 days (**a**–**f**) stained with safranin-O/haematoxylin/fast green (**a**–**c**) or Collagen II antibodies (**e**,**f**). Bioassembled HAC-laden 9.5% GelMA-0.5% HepMA micro-spheres (**g**,**h**) stained with Calcein AM (live cells, green) and Propidium Iodide (dead cells, red) (**g**) or DAPI (blue) and Aggrecan (purple) antibodies (**h**) after 35 days culture in chondrogenic differentiation media). (**B**) The modular capability of the bioassembly platform was used to build biphasic, graduated, and assorted structures of mesenchymal stromal cells (hMSCs) and human nasal chondrocytes (hNCs). (**A**), reproduced with permission from [24]; (**B**), reproduced with permission from [120].

The second group of modular strategies described in this review uses hybrid bioprinting approaches involving the integration of cell-laden microspheres and/or tissue spheroids within the pores of a CAD scaffold made of a thermoplastic synthetic polymer (Figure 5) [24,99,117,118,119,120].

The skeleton structure of the thermoplastic polymer, typically PCL, is used to minimize the forces that the upper layers of the bioink impart on the underlying layers, thereby stabilizing the size and structure of the sample. Woodfield and co-workers used this strategy to fabricate hybrid tissue-engineered constructs for cartilage and osteochondral tissue [99,118,120]. A similar approach using tissue spheroids made of two different cell populations was used to replicate the zonal organization of chondrocytes in native cartilage tissue [117]. Aspects of the printing process that could cause the structure of the spheroid to be compromised during processing have been addressed in the literature. In order to bioassemble the cell-laden modules into a pre-printed and mechanically stable thermoplastic scaffold lattice, the shape fidelity of the modules should be carefully designed to match lattice pores size, while automated module handling must ensure the maintenance of the cell viability and specific differentiation capacity [24,99]. The automated bioassembly of tissue spheroids using fluidic handling and bioprinters is extremely advantageous if compared with manual placement, as it may allow to develop completely automated 3D in vitro tissue models for medium- or high-throughput screening [121]. Modules automation is therefore a critical issue of this approach, as it requires the combination of a singularisation module capable of delivering individual micro-tissues to an injection module, for module insertion within the cages of a 3D plotted scaffold [24]. CAD-AM processes can also be assisted by computational approaches to simulate and predict tissue growth in porous scaffolds as a function of pore geometry and fluid flow [122], modelling tissue growth in 3D printed scaffolds [123] and simulating the physiological process of tissue healing under the effect of controlled biomolecules delivery [124]. The integration of computational methods with 3D printing techniques may also allow for optimizing scaffold design and manufacture [125], and predicting scaffold properties as a function of building blocks composition and spatial assembly for the reduction of experimental iterations [126].

## 4. Conclusions and Future Outcomes

Over the past decades, the concept of TE scaffold has evolved dramatically, starting from a structural support for cell growth and tissue spacer, to the current concept of a cell instructive platform capable of controlling and guiding cellular processes involved in new tissue morphogenesis. The use of engineered cell-free and/or cell-laden micromodules together with CAD-AM processes is an emerging field that holds great promise in TE applications. Technological advancements in microscaffolds design and cell processing have enabled the fabrication of tissue-specific micromodules for applications such as cartilage tissue or vascularized bone. These modules were bioprinted, alone or within the cage of thermoplastic polymers, to study, among others, the development of anisotropic tissue and the role of cell co-culture parameters on tissue formation [120]. Unlike tissue spheroid approaches, engineered microscaffolds can ensure enhanced mechanical stability and cell survival during bioprinting. Furthermore, cells within microscaffolds respond to biomechanical and biochemical cues presented within their surrounding synthetic environment.

Despite advancements in micromodules bioprinting and integration within complex 3D platforms, there remained significant challenges in TE scaffolds. To shed light on the mechanisms involved in cell/material interaction in 3D microenvironments, and to allow scaffold translation from bench to the bedside, TE requires translating 2D cell culture knowledge to 3D micromodules and, finally, to CAD-AM scaffolds. The study of cell function in response to isolated biophysical and biochemical components within biomimetic scaffolds is mandatory, and it requires new materials capable of triggering, even at nanometric size scale, scaffold mechanical properties, degradation, and biomolecule release following a cell and tissue demanding strategy. Advancement in automated micro- and nano-positioning and printing techniques are also needed to improve printing accuracy, to speed up building blocks manipulation, and to reduce fabrication times and increase the scaffold size for effective in vivo testing [127]. The composition, microstructural properties, and physical properties, such as mechanical strength, and surface roughness of the AM scaffolds are key to the design of the process. The material deposition mechanisms are critical towards achieving the desired geometry, resolution, and throughput. Another important issue of bioprinting techniques is the integration of a functional vasculature within a fabricated bioconstruct. In fact, the scalability of scaffolds and bioprinted constructs for applications such as bone implants is limited. Even if the co-culture of endothelial cells and stem cells is a promising approach to promote vascularization [115], significant efforts are needed to achieve stable and perfusable vascular networks within bioconstructs obtained from patterned micromodules. In this context, the standardization of micromodule fabrication in a high-throughput process and the development of lab-on-chip devices for cell growth and testing under the effect of controllable stimuli will be necessary.

Programming the biophysical and biochemical properties of the scaffold focusing on the initial stage of tissue development (e.g., choosing surface activation and drug release depending on initial cell adhesion and ECM deposition or defining growth factor release to attract blood vessels into the scaffold pores) was the standard approach in the past decades. However, scaffold-mediated tissue regeneration requires synchronizing material properties and drug release with developmental stages of new tissue growth, that occur over a period of months to years. To adapt the microenvironmental cues following cell and tissue requirements, the next generation of ECM-mimicking scaffolds must incorporate sensing and actuating molecules within its structure while being supported by techniques for live cell and tissue imaging. In this context, super resolution microscopy and probe chemistries for single-cell detection may enable the live analysis of critical biomarkers, such as cell metabolism and proliferation in 3D scaffolds during tissue growth [128,129,130,131]. Additionally, there will be the need for cellular niche models with the controllable dynamic variation of biophysical and biochemical features, and the capability to measure biomechanical signals at a microscale resolution in a non-invasive and possibly wireless way [132,133,134]. All together, these technological advancements hold the promise that, in a near future, scaffold-based approaches can significantly progress towards clinical implementation and personalized medicine, since they offer the possibility to monitor and guide in vivo tissue regeneration.

## Data Availability

No new data were created or analyzed in this study. Data sharing is not applicable to this article.
